# Exploring Behavioral Risk Factors for Non-communicable Diseases Among Undergraduate Medical Students in Western Gujarat: A Cross-Sectional Study

**DOI:** 10.7759/cureus.49188

**Published:** 2023-11-21

**Authors:** Yogesh M, Nency Kagathara, Rohit Ram, Swati Misra, Jimmy Kagathara

**Affiliations:** 1 Community Medicine, Shri M. P. Shah Government Medical College, Jamnagar, IND; 2 Department of Medicine, Zydus Medical College and Hospital, Dahod, IND; 3 Preventive Medicine, Shri M. P. Shah Government Medical College, Jamnagar, IND

**Keywords:** non-communicable diseases (ncds), stress, obesity adolescent anthropometry, medical school students, behavioral risk factors

## Abstract

Background

The burden of morbidity and death caused by non-communicable diseases (NCDs) such as cancer, diabetes, and cardiovascular disease is a significant global health concern influenced by modifiable behavioral risk factors. In India, the burden of NCDs is particularly high, with medical college students being a vulnerable population. This study aims to bridge the knowledge gap by investigating the prevalence and patterns of behavioral risk factors for NCDs among medical college students.

Methodology

A cross-sectional study was conducted on medical students in Gujarat. Risk factors for NCDs were assessed using various tools, including the General Health Questionnaire-12 (GHQ-12) for stress, the International Physical Activity Questionnaire-Short Form (IPAQ-SF) for physical activity, the Pittsburgh Sleep Quality Index (PSQI) for sleep quality, the body mass index (BMI) for obesity, and dietary factors. The chi-square test was employed as a statistical tool to determine the association between socio-demographic variables and various risk factors. A p-value of <0.05 was considered statistically significant.

Results

Among the 400 students surveyed, the prevalence of single behavioral NCD risk factors was as follows: 248 (62%) reported stress (GHQ-12), 215 (54%) experienced poor sleep quality (PSQI), 251 (63%) had low levels of physical activity (IPAQ), 339 (85%) had inadequate fruit and vegetable intake, 97 (24%) consumed extra salt during meals, 163 (41%) were overweight or obese, and 189 (47%) had three or more risk factors for NCDs. In bivariate logistic regression analysis, factors such as age, male gender, urban residence, hostel stay, and lower socioeconomic status were found to be statistically significant (p < 0.05).

Conclusion

This study reveals an alarming failure of medical colleges to positively influence students' health behaviors, despite their medical knowledge. The high rates of inactivity, stress, poor diet, and obesity among students demonstrate the curriculum's inability to instill preventative lifestyle practices. This omission in training compromises students' own health and their ability to counsel patients on NCD prevention. Urgent reform is needed to integrate health promotion into the curriculum, providing a supportive campus culture focused on wellness. By overlooking students' behaviors, medical colleges gravely disserve these future providers. This evidence compels curriculum reform to develop exemplary physician role models for NCD prevention.

## Introduction

Non-communicable diseases (NCDs) have emerged as a significant global health concern, accounting for a substantial burden of morbidity and mortality worldwide. These chronic diseases, which encompass cardiovascular diseases, diabetes, and various cancers, are primarily influenced by modifiable behavioral risk factors. While NCDs are commonly associated with older populations, recent evidence indicates that the prevalence of these risk factors among young adults, particularly medical college students, is alarmingly high [1-3].

Globally, the prevalence of behavioral risk factors among medical college students is a cause for concern. Studies conducted in various countries have reported alarming rates of sedentary behavior, physical inactivity, unhealthy diets, tobacco and alcohol use, poor stress management, and inadequate sleep within this student population. These factors significantly contribute to the development and progression of NCDs, both during their student years and in their future professional lives [4,5].

In India, the burden of NCDs is notably high, with the country bearing a substantial proportion of the global NCD burden. Among medical college students in India, behavioral risk factors make a significant contribution to this burden.

Despite the global and local evidence, there remains a limited understanding of the specific prevalence rates and patterns of behavioral risk factors among medical college students in India. This study aims to bridge this knowledge gap by investigating the prevalence and patterns of behavioral risk factors associated with NCDs among medical college students.

## Materials and methods

The present cross-sectional study was conducted on undergraduate medical students at Shri M. P. Shah Government Medical College, Jamnagar, Gujarat, between January 2023 and April 2023. The sample size was calculated using the single proportion formula: Z2α/2​P(1−P)/d2, where (Z) represents the 95% confidence interval (1.96), (d) is the absolute error (5%), (n) is the sample size, (P) is the estimated proportion, and Zα/2​ is the critical value (with (P=16%), based on a reference study [6]).

So, 3.84×16×84/16=320. The inclusion criterion was being present in class at the time of recruitment and providing consent to participate. Those who did not give consent were excluded. After assessing eligibility, students were selected randomly, resulting in a total of 400 medical students (150 from the first year, 150 from the second year, and 100 from the final year) being enrolled in this study.

Participation was voluntary, and a self-administered questionnaire was used, which included the General Health Questionnaire-12 (GHQ-12), Pittsburgh Sleep Quality Index (PSQI), International Physical Activity Questionnaire-Short Form (IPAQ-SF), dietary assessment, and weight and height measurements.

Current mental health was evaluated using the GHQ-12. Participants were required to indicate whether they had experienced specific symptoms, with each item rated on a four-point scale: "not at all," "no more than usual," "somewhat more than usual," or "much more than usual." The (0-1-2-3) scoring technique was frequently used to predict the likelihood that the item may cause stress. The possible total scores ranged from 0 to 36, with cases considered to exist when total scores exceeded 12 [7].

The International Physical Activity Questionnaire (IPAQ) is a reliable and valid tool used to assess the level of physical activity. The International Physical Activity Questionnaire-Short Form (IPAQ-SF) was employed to measure the degree and type of physical activity as a predictive variable. This self-report questionnaire was primarily developed to measure physical activity in adults aged 15 to 69 [8].

The IPAQ-SF scoring system assigns "light intensity" activities, such as walking, as well as "moderate" and "vigorous" intensity activities, the following MET values: 3.3 METs, 4.0 METs, and 8.0 METs, respectively. MET stands for Metabolic Equivalent of Task and is a measure of the energy cost of physical activities, used to express the intensity of physical activities. One MET is defined as the energy expenditure for sitting quietly, which is equivalent to a caloric consumption of 1 kcal/kg/hour [8].

The Pittsburgh Sleep Quality Index (PSQI) was used to assess insomnia symptoms among participants. It is a validated instrument that measures sleep quality and disturbances over a one-month period. The index consists of 19 self-rated questions, and the global PSQI score provides an overall measure of sleep quality. Higher scores indicate poorer sleep quality [9,10].

Inadequate fruit and vegetable intake was defined as less than five servings per day (<400 grams) [11].

Regarding socioeconomic status classification, the BG Prasad classification of socio-economic scale based on per-capita income was used. The upper class and upper middle-class categories were merged into the "upper class," while the middle class, lower middle-class, and lower class were merged into the "lower class" [12].

Weight and height were used to calculate the BMI of each student using the formula BMI = weight (in kg) / height (in m^2). BMI is also referred to as the Quetlet Index [13]. The BMI classification is mentioned in Table 1.

**Table 1:** Classification of BMI among participants The BMI cut-off classification used was the Asian Cut-Off Classification [14]. BMI: body mass index

Classification	BMI cut-off
Underweight	<18.5
Normal:	18.5-22.9
Overweight	>23
At Risk:	23-24.9
Obese	≥25
Class-1	25-29.9
Class-2	>30

Written informed consent was obtained from all the participants. This study was conducted after obtaining approval from the Institutional Ethical Committee (Reference: 125/05/2021).

Statistical analysis

It involved entering the collected data into a Microsoft Excel (Microsoft Corporation, Redmond, Washington, United States) spreadsheet, and statistical analysis was performed using IBM SPSS Statistics for Windows, Version 26 (released 2019; IBM Corp., Armonk, New York, United States). The frequency of variables and risk factors was calculated in percentages. The results were summarized in tables in terms of proportions and percentages. The chi-square test of significance was used to determine the association between the presence of risk factors and various socio-demographic variables. A p-value < 0.05 was considered statistically significant.

## Results

The present cross-sectional study was conducted among undergraduate medical students at one of the most prestigious medical colleges in Gujarat. The mean age of the students was 20.12 ± 1.5 years. Out of all the students, 185 (46%) were male, while 215 (54%) were female. The majority of them, 238 (69%), hailed from urban areas, and most, 297 (74%), were residing in hostels. Regarding the medium of study, 214 (54%) students belonged to the English medium, and the majority, 245 (61%), were vegetarians. In terms of socio-economic class, 246 (62%) students were classified as belonging to the upper socio-economic class (Table 2).

**Table 2:** Socio-demographic variables of participants

	First year (N = 150) No. (%)	Second year (N = 150) No. (%)	Third year (N = 100) No. (%)	Total (N = 400) No. (%)
Age	18.4±1.061	19±0.97	20±1.4	20.12±1.5
Gender				
Boy	62(56%)	65(44%)	58(58%)	185(46%)
Girl	88(44%)	85(56%)	42(42%)	215(54%)
Residence				
Rural	61(41%)	54(36%)	9(9%)	124 (31%)
Urban	89(59%)	96(64%)	91(91%)	238(69%)
Stay				
Hostel	118(84%)	89(59%)	90(90%)	297 (74%)
Home	32(16%)	61(41%)	10(10%)	103 (26%)
Medium of study				
English	82(56%)	86(65%)	46(40%)	214 (54%)
Non-English	68(46%)	64(32%)	54(54%)	186 (46%)
Diet				
Veg	131(87.3%)	59(39%)	55(55%)	245 (61%)
Non-veg	19(12.6%)	91(61%)	45(45%)	155(39%)
Socio-economic class				
Upper class	107(71)	106(70.6)	33(33%)	246 (62%)
Lower class	43(28.6)	44(29.3)	67(67%)	154 (38%)

Among all the students, according to the General Health Questionnaire-12 (GHQ-12), stress was present in 248 (62%) of them. There was no significant association found between the presence of stress and the year of study (p = 0.15). The mean Pittsburgh Sleep Quality Index (PSQI) score was 7.7 ± 2.63. Out of all the students, approximately 215 (54%) had poor sleep quality, but no significant association was found between the quality of sleep and the year of study (p = 0.29). About 251 (63%) of students had a level of physical activity according to the International Physical Activity Questionnaire (IPAQ), but there was no significant association found between the level of physical activity and the year of study (p = 0.71). Of all the students, 339 (85%) had inadequate intakes of fruits and vegetables, and about 97 (24%) of them added extra salt to their meals. In the present study, about 94 (23.5%) of students were obese, and around 69 (17%) were overweight, but no significant association was found between obesity and the year of study (Table 3).

**Table 3:** Prevalence of behavioral risk factors among medical students p<0.05*-significant, analysis was done by chi-square GHQ-12: General Health Questionnaire 12; PSQI: Pittsburgh Sleep Quality Index; IPAQ: International Physical Activity Questionnaire

Behavioral risk factor	First year (N = 150) No. (%)	Second year (N = 150) No. (%)	Third year (N = 100) No. (%)	Total (N = 400) No. (%)	P value
Stress according to General Health Questionnaire 12 (GHQ-12)					
Stress	98 (65.33)	96 (64)	54 (54)	248 (62)	0.15
No stress	52 (34.67)	54 (36)	46 (46)	152 (38)	
Pittsburgh Sleep Quality Index (PSQI) score	6.6±1.1	7.2±0.97	6.9±1.4	7.7±2.63	
Sleep Quality Score according to PSQI (Score ≥6)					
Poor sleep quality (Score ≥6)	75 (50)	80 (53.33)	60 (60)	215 (53.75)	0.29
Good sleep quality (Score <6)	75 (50)	70 (46.67)	40 (40)	185 (46.25)	
Physical activity according to the International Physical Activity Questionnaire (IPAQ)					
Low level of physical activity	87 (58)	80 (53.34)	56 (56)	251 (62.75)	0.71
Moderate + vigorous level of physical activity	63 (42)	70 (46.67)	44 (44)	177 (37.25)	
Fruits and vegetable intake					
Inadequate	128 (85.33)	131 (87.33)	80 (80)	339 (84.75)	0.27
Adequate	22 (14.67)	19 (12.67)	20 (20)	61 (15.25)	
Add extra salt during meals					
Yes	34 (22.67)	37 (24.67)	26 (26)	97 (24.25)	0.82
No	116 (77.33)	113 (75.33)	74 (74)	303 (75.75)	
Body mass index classification					
Underweight (< 18.5)	30 (20)	28 (18.67)	16 (16)	74 (18.5)	0.87
Normal (18.5-22.99)	58 (38.67)	62 (41.33)	43 (43)	163 (40.75)	
Overweight (23 – 24.99)	27 (18)	28 (18.66)	14 (14)	69 (17.25)	
Obese (≥25)	35 (23.33)	32 (21.33)	27 (27)	94 (23.50)	

Out of the 400 students, three or more non-communicable disease risk factors were present in 189 (47.25%) students. A higher presence of three or more risk factors was found in the >20-year-old age group, which is statistically significant (p-value < 0.001). A greater number of males had three or more risk factors for NCDs than females, which is statistically significant (p-value < 0.001). Urban residents had a higher likelihood of having three or more risk factors compared to rural residents (p-value = 0.038). Similarly, students staying in hostels were more associated with three or more risk factors for NCDs compared to students staying at home (p-value = 0.047). Students with lower socioeconomic status had a higher likelihood of having three or more risk factors compared to those with higher socioeconomic status (p-value < 0.001) (see Table 4).

**Table 4:** Association between various socio-demographic characteristics and behavioral risk factors of NCD p<0.05*-significant,p<0.01**-highly significant.

Variables	Presence of more than or equal to three risk factors			P value
	Yes, N (%) (N = 189)	No, N (%) (N = 211)	Total N (%) (N = 400)	
Age				
≤20 years	76 (29)	184 (71)	260 (65)	<0.001**
> 20 years	113 (81)	27 (19)	140 (35)	
Gender				
Male	122 (66)	63(34)	185 (46)	<0.001**
Female	67 (31)	148(69)	215 (54)	
Residence				
Urban	140 (51)	136 (49)	276 (69)	0.038*
Rural	49 (40)	75 (60)	124 (31)	
Stay				
Hostel	149 (50)	148 (50)	297 (74)	0.047*
Home	40 (39)	63 (61)	103 (26)	
Socio-economic status				
Lower	127 (82)	27 (18)	154 (39)	<0.001**
Upper	62 (25)	184 (75)	246 (61)	

## Discussion

The present cross-sectional study was conducted among undergraduate medical students. In our study, according to the General Health Questionnaire 12 (GHQ-12), stress was present in 248 (62%) of students. This finding is well correlated with the study conducted by Kazmi et al. [15].

In our study, we found that around 54% of students had poor sleep quality (with a score of ≥6), according to the Pittsburgh Sleep Quality Index. This finding aligns well with the study conducted by Ghrouz A. K. et al. [16].

In the present study, it was found that 62.75% of students had poor physical activity according to the IPAQ score. This percentage was lower compared to Rustagi et al. [17], which reported 88% poor physical activity, but higher compared to other studies conducted by Shrivastav et al., Shenkin et al., and Pengpid et al., which reported poor physical activity ranging from 31% to 50% [18,19,20].

In the present study, a total of 84.75% of students reported having an inadequate intake of fruits and vegetables. This figure was similar to the findings of Pengpid et al. [20], but higher compared to studies conducted by Hadaye & Dass [21] and Ricardo et al. [22]. These findings collectively emphasize the need for targeted interventions to improve dietary behaviors and promote the consumption of fruits and vegetables among medical college students.

Adding extra salt during meals was reported by 24.25% of students in the present study, which is in line with a study conducted in Gujarat, which reported 26% of students adding extra salt [23]. However, 53% of students were found to add extra salt in a study conducted in Delhi [17], and around 78% in Kerala [24]. These findings underscore the need for targeted interventions to raise awareness about the harmful effects of excessive salt consumption and promote the adoption of healthier dietary practices among medical college students.

Around 23.5% of students were found to be obese in the present study, which was higher compared to similar studies in India that showed a prevalence of obesity ranging from 1.5% to 16.5%. It's worth noting that in some of these studies, the BMI cut-off used was based on the WHO classification (BMI > 30 kg/m^2), while in our study, the Asian BMI cut-off (BMI > 25 kg/m^2) was used [18,20,21,24,25]. Additionally, 17.25% of students were found to be overweight or pre-obese. However, a wide variation in overweight prevalence was seen in previous studies in India, ranging from 8% to 47% [18,20,21,24,25]. a Such high prevalence of overweight and obesity across various regions in India, including Uttar Pradesh, Mumbai, Kerala, and Wardha, underscores the need for comprehensive strategies to prevent and manage obesity among medical college students [18,21,24,25].

In the present study, it was found that individuals older than 20 years were more likely to have three or more risk factors for NCDs. A previous study, including students from 24 countries, also reported that increasing age is positively associated with a higher risk of NCD risk factors [26].

In our study, females reported a higher likelihood of having three or more risk factors compared to males, which is also consistent with other studies [26,27,28].

Students residing in urban areas were more likely to have three or more risk factors for NCDs compared to those in rural areas, which is also consistent with findings by Mathur et al. and Thakur et al., suggesting the need to focus more on urban localities for education and awareness regarding these risk factors [27,29].

From the present study, we can conclude that medical students exhibit high levels of stress according to the GHQ-12 scale, poor sleep quality according to the Pittsburgh Sleep Quality Index score and poor physical activity according to the International Physical Activity Questionnaire (IPAQ). Overall, the findings of this study highlight the prevalence of inadequate fruit and vegetable intake, excessive salt consumption, and obesity among medical college students. These findings are consistent with previous studies conducted among similar populations, underscoring the need for targeted interventions to promote a healthy diet and lifestyle.

Recommendations

Implement Targeted Interventions

Develop and implement targeted interventions that focus on promoting healthy behaviors and lifestyle choices among medical college students. These interventions can include nutrition education programs, stress management workshops, and physical activity initiatives.

Enhance Campus Resources

Improve the availability of healthy food options on college campuses, such as by increasing the accessibility of fruits, vegetables, and nutritious meals. Additionally, provide adequate recreational facilities and spaces for physical activity to encourage students to engage in regular exercise.

Integrate Health Promotion Into the Curriculum

Include health promotion and teaching on NCD prevention in the medical curriculum. This will ensure that future healthcare professionals have the knowledge and skills to promote healthy behaviors among their patients and themselves.

Foster a Supportive Environment

Create a supportive environment within medical colleges that emphasizes the importance of healthy behaviors. This can be achieved by establishing wellness committees, organizing health campaigns, and providing counseling services to address stress and mental health concerns.

This study reveals an alarming failure of medical colleges to positively influence students' health behaviors, despite their knowledge. The high rates of inactivity, stress, poor diet, and obesity among students demonstrate the curriculum's inability to instill preventative practices. This omission compromises students' own health and ability to counsel patients. Urgent reform is needed to integrate health promotion into the curriculum, providing a supportive campus culture focused on wellness. Crucially, training must equip students with techniques to motivate positive behavior change in patients for NCD prevention. By overlooking students' behaviors, medical colleges gravely disserve these future providers. This evidence compels curriculum reform to develop exemplary physician role models and provide skills to influence patients’ lifestyle practices, enabling the prevention and management of non-communicable diseases.

Limitations

Despite the valuable insights gained from this study, several limitations should be acknowledged.

Sample Representativeness

The findings may not be generalizable to all medical college students due to the use of convenience sampling within a specific institution. The sample may not fully represent the diverse characteristics and backgrounds of medical students in other settings.

Self-Reported Data

The study relied on self-reported data, which may be subject to recall bias or social desirability bias. Participants might have underreported or overreported their behaviors, potentially affecting the accuracy of the prevalence estimates.

Cross-Sectional Design

The cross-sectional design limits the ability to establish causal relationships between the risk factors and demographic/lifestyle factors. Longitudinal studies would provide more robust evidence of the associations and trends over time.

Single Institution Focus

This study was conducted within a specific medical college, and the findings may not necessarily reflect the situation in other institutions or regions. Therefore, caution should be exercised when extrapolating the results to broader populations.

Despite these limitations, this study provides valuable insights into the prevalence and associations of behavioral risk factors among medical college students, laying the groundwork for future research and interventions in this population.

## Conclusions

The concerningly high prevalence of behavioral risk factors for non-communicable diseases among medical students, as demonstrated in this study, represents a tragic failure of the medical college curriculum and environment to positively shape the lifestyle behaviors of these future physicians. Despite their extensive medical knowledge, the students' poor physical activity levels, high stress, unhealthy diets, and obesity rates underscore the curriculum's inability to translate knowledge into preventative behaviors. This glaring omission in medical training imparts a long-lasting detriment, harming the health of the students themselves and compromising their ability to effectively counsel and motivate patients on lifestyle changes for NCD prevention in the future. Urgent reform is imperative to foster a supportive campus culture and learning climate focused on instilling healthy lifestyle practices among students. Integrating health promotion activities into the curriculum and providing resources to reduce stress and facilitate wellness are crucial first steps. By overlooking the need to influence students' behaviors, medical colleges are doing a major disservice to these future healthcare providers and their patients. This study provides compelling evidence of the need to radically transform training to create exemplary role models for NCD prevention, rather than mediocre ones.

## References

[1] Singh AK, Maheshwari A, Sharma N, Anand K (2006). Lifestyle associated risk factors in adolescents. Indian J Pediatr.

[2] Sreeramareddy CT, Shankar PR, Binu VS, Mukhopadhyay C, Ray B, Menezes RG (2007). Psychological morbidity, sources of stress and coping strategies among undergraduate medical students of Nepal. BMC Med Educ.

[3] Kaur P, Rao SR, Radhakrishnan E, Ramachandran R, Venkatachalam R, Gupte MD (2011). High prevalence of tobacco use, alcohol use and overweight in a rural population in Tamil Nadu, India. J Postgrad Med.

[4] Peng P, Hao Y, Liu Y (2023). The prevalence and risk factors of mental problems in medical students during COVID-19 pandemic: a systematic review and meta-analysis. J Affect Disord.

[5] Al-Farsi OA, Al-Farsi YM, Al-Sharbati MM, Al-Adawi S (2016). Stress, anxiety, and depression among parents of children with autism spectrum disorder in Oman: a case-control study. Neuropsychiatr Dis Treat.

[6] NCD Countdown 2030 collaborators (2018). NCD Countdown 2030: worldwide trends in non-communicable disease mortality and progress towards Sustainable Development Goal target 3.4. Lancet.

[7] Gnambs T, Staufenbiel T (2018). The structure of the General Health Questionnaire (GHQ-12): two meta-analytic factor analyses. Health Psychol Rev.

[8] Papathanasiou G, Georgoudis G, Georgakopoulos D, Katsouras C, Kalfakakou V, Evangelou A (2010). Criterion-related validity of the short International Physical Activity Questionnaire against exercise capacity in young adults. Eur J Cardiovasc Prev Rehabil.

[9] Buysse DJ, Reynolds CF, Monk TH, Berman SR, Kupfer DJ (1989). The Pittsburgh Sleep Quality Index: a new instrument for psychiatric practice and research. Psychiatry research.

[10] Grandner MA, Kripke DF, Yoon IY, Youngstedt SD (2006). Criterion validity of the Pittsburgh Sleep Quality Index: investigation in a non-clinical sample. Sleep Biol Rhythms.

[11] WHO/FAO. WHO/FAO. (2023). Diet, Nutrition and the Prevention of Chronic Diseases: Report of a Joint WHO/FAO Expert Consultation. https://www.who.int/publications-detail-redirect/924120916X.

[12] Mangal A, Kumar V, Panesar S, Talwar R, Raut D, Singh S (2015). Updated BG Prasad socioeconomic classification, 2014: a commentary. Indian J Public Health.

[13] WHO Expert Consultation (2004). Appropriate body-mass index for Asian populations and its implications for policy and intervention strategies. Lancet.

[14] Dudeja V, Misra A, Pandey RM, Devina G, Kumar G, Vikram NK (2001). BMI does not accurately predict overweight in Asian Indians in northern India. Br J Nutr.

[15] Shaikh BT, Kahloon A, Kazmi M, Khalid H, Nawaz K, Khan N, Khan S (2004). Students, stress and coping strategies: a case of Pakistani medical school. Educ Health (Abingdon).

[16] Ghrouz AK, Noohu MM, Manzar MD, Bekele BB, Pandi-Perumal SR, Bahammam AS (2021). Short-term insomnia symptoms are associated with level and not type of physical activity in a sample of Indian college students. J Prev Med Hyg.

[17] Rustagi N, Taneja DK, Mishra P, Ingle GK (2011). Cardiovascular risk behavior among students of a medical college in Delhi. Indian Journal of Comm Med.

[18] Srivastava A, Sharma M, Gupta S, Saxsena S (2013). Epidemiological investigation of lifestyle associated modifiable risk factors among medical students. National Journal of Medical Research.

[19] Martin A, Saunders DH, Shenkin SD, Sproule J (2014). Lifestyle intervention for improving school achievement in overweight or obese children and adolescents. Cochrane Database Syst Rev.

[20] Pengpid S, Peltzer K (2019). Prevalence and correlates of behavioral non-communicable diseases risk factors among adolescents in the Seychelles: results of a national school survey in 2015. Int J Environ Res Public Health.

[21] Hadaye S, Dass R (2021). Assessment of preventable risk factors of cardiovascular diseases among junior college students: a cross-sectional study. Indian Journal of Comm Med.

[22] Ricardo CZ, Azeredo CM, Machado de Rezende LF, Levy RB (2019). Co-occurrence and clustering of the four major non-communicable disease risk factors in Brazilian adolescents: analysis of a national school-based survey. PLoS One.

[23] Puwar T, Saxena S, Yasobant S, Savaliya S (2018). Noncommunicable diseases among school-going adolescents: a case study on prevalence of risk factors from Sabarkantha district of Gujarat, India. Indian Journal of Comm Med.

[24] Sarma PS, Sadanandan R, Thulaseedharan JV (2019). Prevalence of risk factors of non-communicable diseases in Kerala, India: results of a cross-sectional study. BMJ Open.

[25] Kumar R (2015). Anthropometric and behavioral risk factor for non-communicable diseases: a cluster survey from rural Wardha. Indian J Public Health.

[26] Pengpid S, Peltzer K (2021). Prevalence and correlates of multiple behavioural risk factors of non-communicable diseases among university students from 24 countries. J Public Health (Oxf).

[27] Mathur P, Kulothungan V, Leburu S (2021). Baseline risk factor prevalence among adolescents aged 15-17 years old: findings from National Non-communicable Disease Monitoring Survey (NNMS) of India. BMJ Open.

[28] Nair LM, Madhu B, Srinath KM, Gopinath A, Yadav K (2016). Magnitude of behavioural risk factors for cardiovascular diseases among college going young adults (18-25 years) in Mysuru, Karnataka, india. International Journal of Comm Med and Pub Health.

[29] Thakur JS, Jeet G, Nangia R (2019). Non-communicable diseases risk factors and their determinants: a cross-sectional state-wide STEPS survey, Haryana, North India. PLoS One.

